# Biomechanical and Bone Material Properties of Schnurri‐3 Null Mice

**DOI:** 10.1002/jbm4.10226

**Published:** 2019-09-11

**Authors:** Jochen G Hofstaetter, Barbara M Misof, Dallas C Jones, Ruth Zoehrer, Stéphane Blouin, Christiane Schueler, Eleftherios P Paschalis, Reinhold G Erben, Richard Weinkamer, Klaus Klaushofer, Paul Roschger

**Affiliations:** ^1^ 1st Medical Department Hanusch Hospital Ludwig Boltzmann Institute of Osteology at the Hanusch Hospital of WGKK and AUVA Trauma Centre Meidling Vienna Austria; ^2^ Orthopaedic Hospital Vienna Speising Vienna Austria; ^3^ Department of Immunology and Infectious Diseases Harvard School of Public Health Boston MA USA; ^4^ Department of Biomedical Sciences University of Veterinary Medicine Vienna Austria; ^5^ Department of Biomaterials Max Planck Institute of Colloids and Interfaces Potsdam Germany

**Keywords:** Shn3‐NULL MICE; Shn3‐DEFICIENCY; BONE BIOMECHANICS; BONE MATERIAL QUALITY, BONE MINERALIZATION; DENSITY DISTRIBUTION, COLLAGEN CROSS‐LINK RATIO

## Abstract

*Schnurri‐3 (Shn3)* is an essential regulator of postnatal skeletal remodeling. Shn3‐deficient mice (Shn3–/–) have high bone mass; however, their bone mechanical and material properties have not been investigated to date. We performed three‐point bending of femora, compression tests of L3 vertebrae. We also measured intrinsic material properties, including bone mineralization density distribution (BMDD) and osteocyte lacunae section (OLS) characteristics by quantitative backscatter electron imaging, as well as collagen cross‐linking by Fourier transform infrared microspectroscopy of femora from Shn3–/– and WT mice at different ages (6 weeks, 4 months, and 18 months). Moreover, computer modeling was performed for the interpretation of the BMDD outcomes. Femora and L3 vertebrae from Shn3–/– aged 6 weeks revealed increased ultimate force (2.2‐ and 3.2‐fold, *p* < .01, respectively). Mineralized bone volume at the distal femoral metaphysis was about twofold (at 6 weeks) to eightfold (at 4 and 18 months of age) in Shn3–/– (*p* < .001). Compared with WT, the average degree of trabecular bone mineralization was similar at 6 weeks, but increased at 4 and 18 months of age (+12.6% and +7.7%, *p* < .01, respectively) in Shn3–/–. The analysis of OLS characteristics revealed a higher OLS area for Shn3–/– versus WT at all ages (+16%, +23%, +21%, respectively, *p* < .01). The collagen cross‐link ratio was similar between groups. We conclude that femora and vertebrae from Shn3–/– had higher ultimate force in mechanical testing. Computer modeling demonstrated that in cases of highly increased bone volume, the average degree of bone matrix mineralization can be higher than in WT bone, which was actually measured in the older Shn3–/– groups. The area of 2D osteocyte lacunae sections was also increased in Shn3‐deficiency, which could only partly be explained by larger remnant areas of primary cortical bone. © 2019 The Authors. *JBMR Plus* published by Wiley Periodicals, Inc. on behalf of American Society for Bone and Mineral Research.

## Introduction

*Schnurri‐3 (Shn3)*, a member of the ZAS family of zinc finger proteins, has been identified as a potent and essential regulator of postnatal skeletal remodeling.^1^, ^2^ Studies in mice indicate that Shn3 regulates osteoblastic bone formation via ERK (extracellular‐signal regulated kinases) downstream of Wnt signaling.^3^ Further mouse studies suggested that Shn3 might inhibit osteoblast activity directly through Runx‐2, and might control osteoclastic bone resorption indirectly.^2^, ^4^, ^5^ Previous in vitro experiments provided evidence that Shn3–/– calvarial osteoblast's mRNA expression was different from controls; levels of bone sialoprotein and osteocalcin were elevated, whereas levels of alkaline phosphatase mRNA were similar to controls.^5^ Recently, significant increases in Shn3 mRNA expression in the presence of proinflammatory cytokines were shown, suggesting a signal for osteoclast activation in the studied human fibroblast‐like cells.^6^

Shn3‐null mice are born healthy, yet show markedly augmented osteoblastic bone formation,^2^ as well as a reduction in osteoclastic bone resorption,^4^ resulting in a high bone mass phenotype. These mice continue to accrue bone with age and are therefore protected from age‐related bone loss,^4^ as well as loss caused by disuse resulting from muscle paralysis induced via Botox‐induced transient paralysis of calf muscles.^4^ Moreover, inducible knockdown of SHN3 enhances bone mass in adult mice.^3^ These results in mice suggest that the inhibition of Shn3 expression or activity might be a promising target for the development of antiosteoporotic drugs.^3^

Previous studies have shown that in addition to bone mass/volume, several other factors, including bone architecture and inherent bone material properties such as bone mineral and matrix tissue characteristics, play a pivotal role in the overall mechanical competence of bone.^7^, ^8^ Currently, osteoporosis treatments such as antiresorptives (eg, bisphosphonates) or anabolic agents (eg, parathyroid hormone) lead to an increase in bone mass as measured by X‐ray absorptiometry; however, they differ significantly in their effect on bone material properties.^9^, ^10^, ^11^, ^12^ Thus, information about the mechanical performance and the intrinsic bone material properties in the absence of Shn3 is important to obtain so as to accommodate the potential development of an antiosteoporotic intervention based on the inhibition of Shn3.

In the present work, we studied bones from Shn3–/– and WT mice at different ages. Overall mechanical performance was measured using typical biomechanical tests including three‐point bending of femora^13^ and compression tests of vertebrae.^14^ We also investigated bone material properties focusing on the bone matrix mineralization. Mineralization of newly formed healthy bone is a multistep process involving many factors, such as mineral‐binding extracellular matrix proteins and proteoglycans, mineralization‐inhibiting proteins, and matrix vesicles.^15^, ^16^ In view of previous results from in vitro studies previously discussed, it is important to have information on the mineralization of the bone formed under Shn3 deficiency. We used quantitative backscatter electron imaging (qBEI)^8^ to characterize the bone mineralization density distribution (BMDD) and osteocyte lacunae section (OLS) characteristics in long bones. Finally, Fourier transform infrared imaging (FTIRI)^17^ was performed to gain information on enzymatic collagen cross‐links in trabecular bone.

## Materials and Methods

### Samples and sample preparation

The generation of *Shn3*‐deficient mice with a C57BL/6 background followed the institutional guidelines for the care, husbandry conditions, and use of animals (described in detail elsewhere).^(2)^ For biomechanical tests, L3 vertebrae (*n* = 6 from Shn3–/– and *n* = 7 from WT) and femurs (*n* = 6 from Shn3–/– and *n* = 7 from WT) from 6‐week‐old animals were harvested and stored at –80°C before testing.

For bone material analysis, undecalcified femora from 6‐week‐old, 4‐month‐old, and 18‐month‐old male and female WT and Shn3–/– mice were retrieved, fixed in alcohol, and embedded in polymethylmethacrylate (PMMA). Three‐ to four‐micron‐thin longitudinal sections were cut from the samples block for FTIRI analysis. The remaining block was prepared for qBEI by grinding and polishing, and the surface carbon coated as previously described.^8^, ^18^

### Bone biomechanics

The volumetric bone mineral density (vBMD) and the cross‐sectional area of vertebrae and femora from mice aged 6 weeks were measured using pQCT (XCT Research M+ pQCT machine; Stratec Medizintechnik, Pforzheim, Germany). For this purpose, femora and L3 vertebrae were obtained and stored in 70% ethanol after necropsy. In the femur, one slice (0.2‐mm‐thick) in the middiaphysis and three slices in the distal femoral metaphysis located 1.5, 2.0, and 2.5 mm from the articular surface of the knee joint were measured. In the L3 vertebrae, three slices were measured, one in a midtransversal plane and two located 0.5 mm rostral and caudal of the midtransversal plane. The vBMD of the femoral metaphysis and that of the vertebra represents the mean for these slices. Voxel size was 70 μm for all measurements. A threshold of 450 mg/cm^‐3^ was used for detection of trabecular bone, and thresholds of 500 mg/cm^‐3^ and 600 mg/cm^‐3^ were used for measurement of cortical bone in L3 vertebrae and femora, respectively.^14^

Femora and vertebrae from mice aged 6 weeks were loaded to failure as described in detail elsewhere.^14^ A Zwick Z020/TN2A material testing machine (Zwick GmbH & Co KG, Ulm, Germany) utilizing a 1‐kN‐force detector with a force resolution of 0.01 N^14^ was used. Briefly, three‐point bending tests of the femurs were performed under hydrated bone matrix conditions. The distance between the lower supports for the bending tests was 5 mm. In the compression tests, planoparallel vertebral body cylinders of 2.0‐mm height were loaded to failure.

Geometric characteristics of the vertebrae and femora were included in the calculation of the apparent strength. For the latter, the ultimate force of the L3 under compression was normalized to the cross‐sectional area of the L3, whereas the ultimate force under bending was normalized to the areal moment of inertia as described below. We use the term “apparent strength” (σ) as this calculated measure does not consider the void volume within the bone tissue, which is particularly high in the vertebral body. Thus for the vertebra, the apparent strength was calculated as a ratio between the ultimate force *F*_*max*_ and the total cross‐sectional bone tissue area, *CsAr*, ie,

(1) $$\sigma =F_{\max}/\text{CsAr}$$

For the estimation of the apparent strength of the femora, the cross‐sectional cortical area (*Ct.Ar*) and the cortical thickness (*Ct.Th*) obtained from the pQCT were used to calculate the outer (*R*_*outer*_) and inner radius (*R*_*inner*_), assuming that the cross‐section of the femur is a circular ring:

(2) $$R_{\text{outer}}=\frac{\frac{\mathrm{Ct}.\mathrm{Ar}}{\pi }+\mathrm{Ct}.{\mathrm{Th}}^{2}}{2\mathrm{Ct}.\mathrm{Th}}$$

and

(3) $$R_{\text{inner}}=R_{\text{outer}}-\mathrm{Ct}.\mathrm{Th}.$$

Based on the information regarding *R*_*outer*_ and *R*_*inner*_, the areal moment of inertia (*I*), a measure for the bone's resistance to bending, can be formulated:

(4) $$I=\frac{\pi }{4}\left(R_{\text{outer}}^{4}-R_{\text{inner}}^{4}\right)$$

and an estimation of the strength σ can be calculated using the formula given in ref^13^

(5) $$\sigma =\frac{F_{\text{max}}\mathrm{Lc}}{4I}$$

where $\frac{F_{\max}L}{4}$ is the bending moment (with *F*_*max*_ being the ultimate force and *L* the distance between the lower supports) and *c* the distance from the neutral axis to the point of loading (ie, *R*_*outer*_ in this case).

### Quantitative backscattered electron imaging (qBEI)

qBEI is based on the backscatter electron signal from an approximately 1.5‐μm‐ thick bone surface layer. This signal is principally dependent on the average atomic number in the target material and thus related to calcium content in bone (details described previously^18^). A scanning electron microscope (DSM 962; Zeiss, Oberkochen, Germany) equipped with a four‐quadrant semiconductor backscattered electron BE detector was used. The BE‐signal (gray scale) was calibrated using the “atomic number contrast” between carbon (C; Z = 6) and aluminum (Al; Z = 13) as reference materials. Consequently, one gray‐level step corresponds to 0.17 wt% Ca. The calibrated digital images were used for a 2D estimation of microstructure of mineralized (md) bone (mdBV/TV; mineralized bone volume per tissue volume), mdTb.Th (mineralized trabecular thickness), mdTb.N (mineralized trabecular number), and for the BMDD.

**Figure 1:**
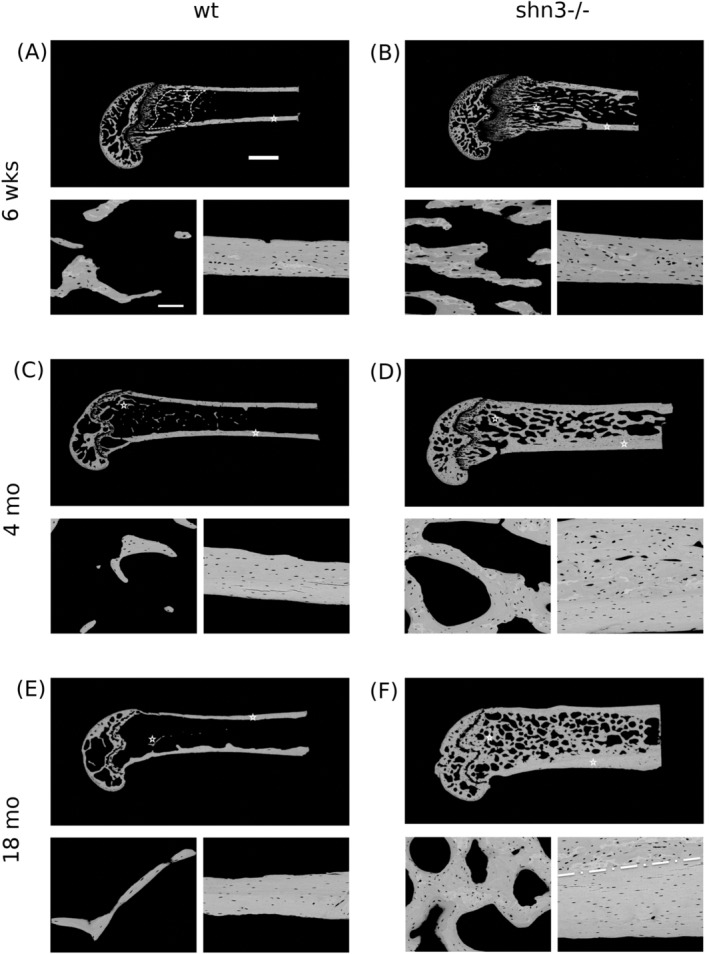
Backscatter electron (BE) overview images of typical examples of longitudinal sections of distal femora from 6‐week‐old (*A*, *B*), 4 month old (*C*, *D*), and 18‐month‐old (*E*, *F*) WT and Shn3 null mice (WT in *A*, *C*, *E*; Shn3–/– in *B*, *D*, *F*). Generally, the images show the tremendous increase in cancellous and cortical bone volume with age in the Shn3‐deficient mice. The region indicated by the dashed line in the overview image in Fig. 1 *A* indicates the region of interest for the analysis of the microstructure of the mineralized bone, and the bar represents 1 mm. Detail images of cancellous and cortical bone obtained at higher magnification are also shown (the asterisks in the overview images show the area of the detail images). The bar in the detail image indicates 100 μm. In the detail image of the Shn3–/– mouse aged 18 months (*F*), the dashed white line indicates the boundary of the remnant area of primary cortical bone (top) from the lamellar cortical bone (bottom). Such two distinct areas can be seen in cortical bone detail images for all Shn3–/– and for the youngest WT. Osteocyte lacunae section morphology is clearly different in primary compared with lamellar cortical bone.

Evaluation of mdBV/TV for the metaphyseal region was performed using a custom‐made automated image analysis routine (NIH Image 1.52, W. Rasband, National Institutes of Health, Bethesda, MD, USA) of qBEI images (Fig. 1A‐F) by discriminating between gray levels of mineralized and not mineralized tissue or embedding material as described elsewhere.^19^ Measurements of mdBV/TV were performed at a distance of 500 to 1500 μm from the growth plate in the sagittal sections (region of interest for this analysis is indicated by the dashed line in Fig. 1 *A*).

**Figure 2:**
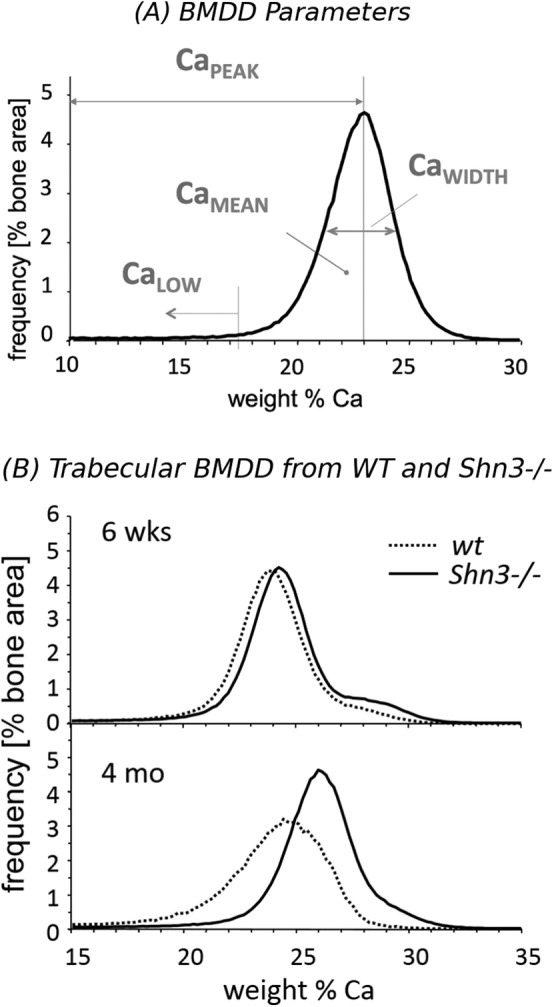
(*A*) Example of a bone mineralization density distribution‐ (BMDD‐) curve showing the calculated BMDD parameters. CaMean = weighted mean calcium concentration; CaPeak = most frequent calcium concentration; CaWidth = full width at half maximum of the BMDD, an index for the heterogeneity of mineralization; CaLow = percentage of bone area having a calcium concentration below 17.68 wt% Ca, representing the percentage of low mineralized lamellar bone undergoing the primary bone mineralization process. (*B*) Measured BMDD from WT and Shn3–/– aged 6 weeks (top) or 4 months groups (bottom). Dotted lines indicate WT, solid lines Shn3–/–.

BMDD measurements were performed in cancellous bone at the metaphyseal region, as well as in cortical bone in the midshaft region. For cancellous BMDD, bone was measured within the metaphysis in each sample (the area of 500 μm adjacent to the growth plate was excluded). Cortical BMDD was obtained from regions as shown in Fig. 1, in the midshaft of both cortices. Calibrated digital BE‐images with a 200× (resolution 1 μm/pixel) nominal magnification were acquired, from which gray‐level histograms (frequency distributions) were derived indicating the percentage of mineralized bone area (corresponding to the number of pixels) with a certain gray level. The following BMDD parameters were calculated (see plotted in the BMDD‐example in Fig. 2 *A*): CaMean, corresponding to the weighted average Ca concentration of the mineralized bone area; CaPeak, which is the most frequently observed Ca concentration; CaWidth, characterized as the width at half‐maximum of the BMDD histogram peak indicating the heterogeneity of mineralization caused by the coexistence of bone areas of different ages and thus of different degrees of mineralization; and CaLow, representing the amount of tissue area mineralized below 17.68 wt% Ca, which is the mineral content achieved in newly formed lamellar human bone during the primary mineralization process.^20^

Additionally, the calibrated qBEI images were also analyzed for 2D‐information about the osteocyte lacunae sections (OLS) characteristics in the cortical bone area.^21^ For this purpose, qBEI images were transformed to binary images using a threshold based on a fixed gray level (corresponding to 5.2 wt% Ca). The OLS were extracted using a minimum and maximum size threshold of 5 μm^2^ and 80 μm^2^, and analyzed based on a custom‐made macro in ImageJ software (version 1.50f; NIH, Bethesda, MD, USA)^22^ as described previously.^21^ The calculated parameters included

$$\mathrm{OLS}-\text{density}\;\left(\frac{\mathrm{nb}.}{{\mathrm{mm}}^{2}}\right)=\frac{\text{number of}\;\mathrm{OLS}\;}{{\mathrm{mm}}^{2}\;\text{mineralized bone area}}$$

$$\mathrm{OLS}-\text{porosity}\;\left(\%\right)=100\times \frac{\mathrm{OLS}\;\text{total area}}{\text{mineralized bone area}+\mathrm{OLS}\;\text{total area}}$$

$$\mathrm{OLS}-\text{area}\;\left({\mathrm{\mu m}}^{2}\right)=\text{mean}\;\mathrm{OLS}\;\text{area}\;\mathrm{per}\;\text{sample}$$

$$\mathrm{OLS}-\text{perimeter}\;\left(\mathrm{\mu m}\right)=\text{mean}\;\mathrm{OLS}\;\text{perimeter}\;\mathrm{per}\;\text{sample}$$

The viability of the cells within the OLS cannot be evaluated by this method.

### Computer modeling of the average mineral content (CaMean)

As previously described,^23^ a mathematical model was applied to the metaphyseal trabecular bone of Shn3–/– mice to simulate the time evolution of the BMDD and CaMean.^23^ The model assumes the mineralization kinetics to be independent of age and genotype. In contrast to previous applications of this model, the trabecular BV/TV was not set constant, but was allowed to increase rapidly with time. The rates of bone deposition and bone resorption were chosen to agree with the measured BV/TV.

### Fourier transform infrared imaging (FTIRI)

FTIRI was used to determine the collagen pyridinoline (pyr)/divalent enzymatic cross‐link ratio (pyr – trivalent/divalent ratio) in metaphyseal cancellous bone tissue of mice aged either 6 weeks or 4 months. All spectroscopic analyses were performed in transmission, on 4‐μm‐thick bone sections, using a Bruker Equinox 55 spectrometer coupled to a mercury cadmium telluride (MCT) focal plane array detector (64 × 64 array) imaged onto the focal plane of an IR microscope (Bruker Hyperion 3000; Bruker Optics, Vienna, Austria). Spectral resolution of 4 cm^−1^ and spatial resolution of approximately 6.3 × 6.3 μm^2^ were used. The instrument was equipped with a 15× objective lens and a motorized stage (±1 μm). FTIRI spectral images (*n* = 3 per sample, each covering an area of 300 × 300 μm^2^ and measured once) were collected in trabecular bone areas and the mean pyr/divalent collagen cross‐link ratios were evaluated. For that, the spectral regions were baseline corrected, and water vapor and polymethylmethacrylate (PMMA) were spectrally subtracted^24^ using Grams 32 (Salem, MA, USA) software. Along with baseline correction, the spectral analysis of the amide I band was performed to isolate underlying peaks (approximately 1660 and 1690 cm^−1^), corresponding to two of the major type I bone collagen cross‐links, namely pyr and divalent ones to calculate the ratio of pyr/divalent.^24^

### Statistical analysis

All measurements were performed with the investigator blinded to the animal groups. Biomechanical data, cross‐sectional areas, and BMDs which were obtained from animals of one specific age were compared using unpaired *t* tests or Mann‐Whitney rank sum tests (if data were not normally distributed). The statistical comparison for histomorphometric structural parameters, BMDD data, OLS‐characteristics, and collagen cross‐link ratios (which were all obtained from mice of different ages) was based on two‐way ANOVA tests (factors genotype and age) with Tukey post hoc comparisons. Statistical analyses were done using SigmaStat 4.0 software (Systat Software, San Jose, CA, USA). The confidence level was set at 95%.

## RESULTS

### Mechanical competence of whole bone

Shn3–/– mice had significantly greater bone ultimate force (F_max_) at both the appendicular and axial skeleton compared with WT mice (Table 1). F_max_ of femurs in three‐point bending was 2.2‐fold (*p* < .001) and L3 vertebral bodies in compression tests was 3.2‐fold (*p* < .01) increased. This increase in mechanical competence was associated with a significant increase in the cortical cross‐sectional area of the femur at midshaft (+50%) and with an increase of cross‐sectional area of the vertebrae (+30%, both *p* = .001) as measured by pQCT before mechanical testing. Considering the contribution of the bone geometry to the measured ultimate force the resulting apparent strength [Equations (1), (5)] was still significantly higher in both, bending (*p* = .01) and compression (*p* = .003) for Shn3–/– compared with WT bone. Additionally, the volumetric bone density was significantly elevated for both skeletal sites (+21% for femur and +30% for vertebra, both *p* < .001; data summarized in Table 1).

**Table 1:** pQCT and Biomechanical Data

Femur			
	WT (*n* = 7)	Shn3–/– (*n* = 6)	*p* Value
vBMD (mg/cm^3^)	531.5 (23.3)	644.1 (31.8)	<.001
CsAr (mm^2^)	1.96 (0.06)	2.51 (0.12)	<.001
Ct.Ar (mm^2^)	0.78 (0.76; 0.81)	1.17 (1.12; 1.38)	.001
Ct.Th (mm)	0.18 (0.17; 0.19)	0.24 (0.23; 0.29)	.001
F_max_ (N)	8.26 (0.96)	18.18 (2.00)	<.001
I (mm^4^)	0.20 (0.19; 0.20)	0.37 (0.33; 0.41)	.001
Apparent Strength σ (MPa)	41.32 (4.46)	55.29 (10.86)	.01

Vertebra L3			
	WT (*n* = 7)	Shn3–/– (*n* = 6)	*p* Value
vBMD (mg/cm^3^)	419 (18.6)	543.3 (35.8)	<.001
CsAr (mm^2^)	1.27 (1.24; 1.46)	1.61 (1.54; 1.67)	.001
Ct.Th (mm)	0.32 (0.01)	0.54 (0.03)	<.001
F_max_ (N)	7.46 (4.69)	23.75 (8.95)	.001
Apparent Strength σ (MPa)	5.67 (3.47)	14.85 (5.96)	.003

Data are mean (SD) or median (25th; 75th percentiles). *n* = number of studied mice. *p* Values are based on *t* test or Mann‐Whitney rank sum test.

vBMD = volumetric bone mineral density; Ct.Ar = cortical area; Ct.Th = cortical thickness; F_max_ = ultimate force under bending or compression; I = areal moment of inertia; apparent strength = ultimate force normalized to I [(under bending, see Equation (5)] or CsAr [under compression, see Equation (1)]; CsAr = total cross‐sectional bone tissue area.

### Bone mineralization density distribution (BMDD)

**Figure 3:**
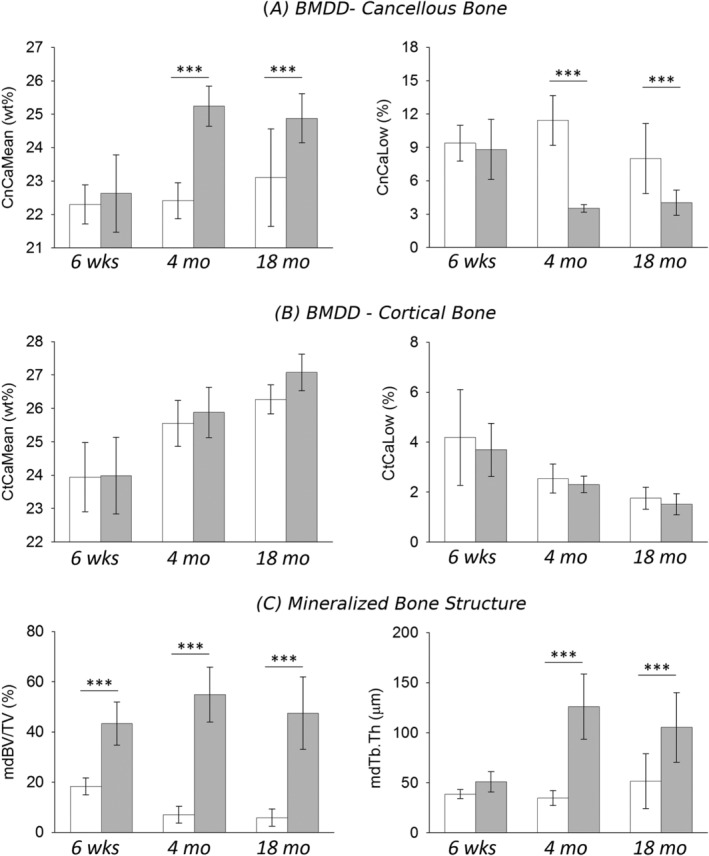
Average degree of mineralization density (CaMean) and the percentage of low mineralized bone areas (CaLow) in (*A*) cancellous and (*B*) cortical bone of the distal femur. (*C*) Microstructure of mineralized bone (mdBV/TV = mineralized bone volume per tissue volume and mdTb.Th = mineralized trabecular thickness) for WT and Shn3–/– mice at different ages. Data are mean (SD), WT in white, and Shn3–/– in gray. *n* = 8 animals per genotype at age 6 weeks, *n* = 6 at 4 months and *n* = 8 at 18 months. *p* < .001 based on Tukey post hoc test following two‐way ANOVA. Only significant differences between Shn3–/– and WT of same age are shown. For all significant differences see Table 2.

Figure 1 shows typical overview qBEI images (one characteristic image per study group) together with examples of detailed images of cancellous and cortical bone for each sample of the investigated longitudinal distal femoral bone. Figure 2 shows the derived BMDD parameters (Fig. 2 *A*) and typical BMDD curves measured in Shn3–/– and WT animals (Fig. 2 *B*). In Fig. 2 *B*, the similarity of the trabecular BMDDs from Shn3–/– and WT at 6 weeks of age and the differences between the genotypes at 4 months are demonstrated. In the full‐grown animals, the BMDD of Shn3–/– is shifted toward higher mineralization. Two‐way ANOVA followed by Tukey post hoc tests revealed an interaction between the factors genotype and age for all trabecular BMDD parameters, indicating that the age development of the trabecular BMDD is different in the two study groups, Shn3–/– and WT (Table 2). Trabecular CaMean was not different at the age of 6 weeks, whereas it was significantly increased at 4 and 18 months in Shn3–/– compared with WT (+12.6% and +7.7%, respectively, both *p* < .001), see also Fig. 3A. At the age of 4 months and 18 months, trabecular CaLow was significantly reduced in Shn3–/– (by –69.2% and –49.4%, respectively, both *p* < .001; Table 2). With increasing age, the Shn3–/– bone material showed significant changes in trabecular CaMean, CaPeak, and CaLow (Table 2); these parameters revealed only minor changes with age in the WT animals (Table 2).

**Table 2:** Bone Mineralization Density Distribution of Trabecular and Cortical Bone From the Femur

		6 Weeks		4 Months		18 Months		Two‐way ANOVA (*p* values)		
		WT (*n* = 8 )	Shn3–/– (*n* = 8)	WT (*n* = 6)	Shn3–/– (*n* = 6)	WT (*n* = 8)	Shn3–/– (*n* = 8)	inter‐action	geno‐type	Age
Trabecular Bone	CaMean	22.30	22.63	22.41	**25.24** a ^,^ d	23.10	**24.88** a ^,^ d	.004	<.001	<.001
	(wt% Ca)	(0.59)	(1.16)	(0.53)	**(0.60)**	(1.45)	**(0.73)**			
	CaPeak	23.40	23.18	24.52	**25.79** c ^,^ d	24.33	**25.84** b ^,^ d	.019	.003	<.001
	(wt% Ca)	(0.61)	(1.27)	(0.59)	**(0.77)**	(1.03)	**(0.71)**			
	CaWidth	3.12	3.25	3.76 e	**3.29** c	3.49	3.57	.035	.395	.005
	(Δwt%Ca)	(0.13)	(0.43)	(0.53)	**(0.16)**	(0.25)	(0.21)			
	CaLow	9.38	8.81	11.43	**3.52** a ^,^ d	7.98 h	**4.04** a ^,^ d	<.001	<.001	<.001
	(% B.Ar)	(1.62)	(2.69)	(2.23)	**(0.35)**	(3.15)	**(1.13)**			
Cortical Bone	CaMean	23.94	23.99	25.55 e	25.88 d	26.27 d	27.08 d ^,^ h	.420	.121	<.001
	(wt% Ca)	(1.04)	(1.14)	(0.69)	(0.75)	(0.43)	(0.55)			
	CaPeak	24.87	24.57	26.23 e	26.51 d	26.82 d	27.49 d	.215	.359	<.001
	(wt% Ca)	(0.86)	(1.08)	(0.77)	(0.75)	(0.43)	(0.59)			
	CaWidth	3.01	3.01	2.92	**3.18** c	3.03	**3.70** a ^,^ d ^,^ g	<.001	<.001	<.001
	(Δwt%Ca)	(0.16)	(0.13)	(0.53)	**(0.14)**	(0.13)	**(0.38)**			
	CaLow	4.19	3.69	2.54 f	2.31 f	1.76 d	1.51 d	.814	.430	<.001
	(% B.Ar)	(1.92)	(1.07)	(2.23)	(0.33)	(0.44)	(0.42)			

Data show mean (SD). *n* = number of studied animals. In bold: data that are significantly different to WT at similar age. Significance by Tukey post hoc comparison following two‐way ANOVA is indicated by superscript letters. CaMean = weighted mean calcium concentration; CaPeak = most frequent calcium concentration; CaWidth = full width at half maximum of the bone mineralization density distribution, an index for the heterogeneity of mineralization; CaLow = percentage of bone area having a calcium concentration below 17.68 wt% Ca, representing the percentage of low mineralized lamellar bone undergoing the primary bone mineralization process; B.Ar = Bone Area.

a *p* < .001.

b *p* < .01.

c *p* < .05 versus WT.

d *p* < .001.

e *p* < .01.

f *p* < .05 versus 6 weeks.

g *p* < .001.

h *p* < .05 versus 4 months (all based on Tukey post hoc comparison).

For cortical BMDD parameters, the two‐way ANOVA comparison did not reveal any significant interaction between factors genotype and age, except for CaWidth (Table 2, Fig. 3B). Post hoc comparison indicated significantly higher cortical CaWidth in Shn3–/– compared with WT at the ages of 4 and 18 months (+8.9%, *p* < .05 and +22%, *p* < .001, respectively; Table 2). The changes in cortical BMDD with age showed an increase in CaMean and CaPeak and a decrease in CaLow in both genotypes (Table 2), whereas CaWidth remained constant in WT and increased in Shn3–/–.

### Relationship between changes in cancellous bone volume and CaMean

**Figure 4:**
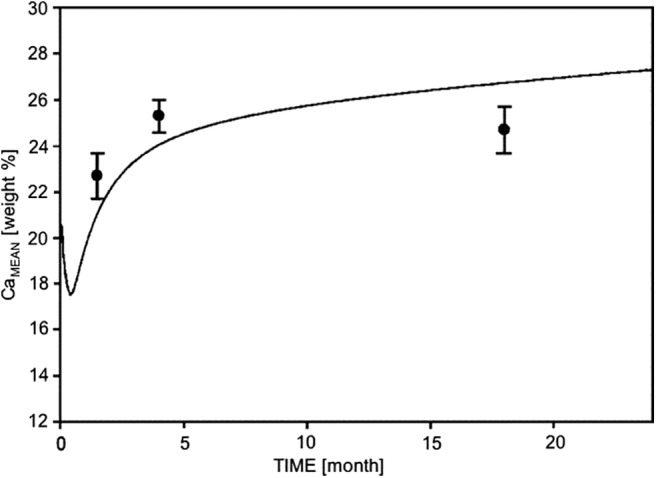
Simulation of time evolution of CaMean (weighted mean calcium concentration), when the trabecular bone volume is increasing with animal age as observed in the Shn3–/– mice. Line: prediction from the computer model; black solid circles: experimental data points. Error bar ±SD.

The mineralized cancellous bone volume of the distal femoral metaphysis was significantly increased in Shn3–/– mice compared with WT. The high bone mass phenotype in Shn3–/– mice was associated with a distinct increase in thickness of mineralized trabeculae as demonstrated in Figs. 1 and 3C. Shn3–/– had a cancellous mdBV/TV of 43% in the youngest age group, which increased up to 55% at 4 months (see Table 3; an overview of this increase can be seen qualitatively also in the images of the longitudinal sections from the mice in Fig. 1). In contrast, WT exhibited mdBV/TV of only 18% at the age of 6 weeks, which declined to 7% at 4 months. Computer modeling of the BMDD^23^ revealed that bone remodeling with a positive net balance leads to an increased CaMean in the long run (Fig. 4). The simulated time course of CaMean shows that the considerable deposition of bone initially causes a decrease of CaMean (at an animal age not considered in this work). The continuation of bone deposition (increase in mdBV/TV) leads to a marked increase in CaMean with age. This time course is in line with the experimentally measured cancellous CaMean values of the Shn3–/– (Fig. 4).

**Table 3:** Microstructure of Mineralized Trabecular Bone at the Metaphysis of the Femur

	6 Weeks		4 Months		18 Months		Two‐way ANOVA (*p* values)		
	WT (*n* = 8 )	Shn3–/– (*n* = 8)	WT (*n* = 6)	Shn3–/– (*n* = 6)	WT (*n* = 8)	Shn3–/– (*n* = 8)	Inter action	geno type	Age
mdBV/TV (% )	18.33	**43.41** a	7.10	**54.91** a ^,^ c	5.87 c	**47.55** a	.003	<.001	.301
	(3.35)	**(8.58)**	(3.35)	**(10.97)**	(3.44)	**(14.37)**			
mdTb.Th. (μm)	38.69	51.04	34.73	**126.1** a ^,^ b	51.60	**105.3** a ^,^ b	<.001	<.001	<.001
	(4.48)	(10.12)	(7.47)	**(32.68)**	(27.37)	**(34.85)**			
mdTh.N (1/mm)	4.74	**8.69** a	1.99 b	**4.41** a ^,^ b	1.19 b	**4.61** a ^,^ b	.136	<.001	<.001
	(0.68)	**(1.79)**	(0.61)	**(0.30)**	(0.61)	**(0.92)**			

Data are mean (SD). *n* = number of studied mice. In bold: data that are significantly different to WT at similar age (based on Tukey post hoc comparison). Significance by Tukey post hoc comparison following two‐way ANOVA is indicated by superscript letters. mdBV = mineralized bone volume per tissue volume; mdTb.Th. = mineralized trabecular thickness; mdTh.N = mineralized trabecular number.

a *p* < .001 versus WT.

b *p* < .001.

c *p* < .05 versus 6 weeks (all based on Tukey post hoc comparison).

### OLS characteristics

Two‐way ANOVA comparison between the 2D‐OLS characteristics as obtained from the qBEI images in cortical bone of the femoral midshaft revealed significant differences for the factors age and genotype (mean and SD, as well as *p* values for the factors are given in Table 4). The interaction between these factors was not significant for all OLS parameters. Post hoc Tukey comparison of the separate study groups showed elevated OLS porosity in Shn3‐deficient mice at the age of 6 weeks (+21%) and 4 months (+33%; both *p* < .05), whereas OLS density was not significantly different between the groups. The OLS area was higher in Shn3‐deficient mice at all ages studied (+16% at 6 weeks, *p* = .001; +23% at 4 months, *p* < .01; and +21% at 18 months, *p* < .01). OLS perimeter was increased in Shn3–/– at the age of 4 months (+7.7%) and 18 months (+8.0%; both *p* < .01).

**Table 4:** Osteocyte Lacunae Sections (OLS) Characteristics of Cortical Bone of the Femur

	6 Weeks		4 Months		18 Months		Two‐way ANOVA (*p* values)		
	WT (*n* = 8 )	Shn3–/– (*n* = 8)	WT (*n* = 6)	Shn3–/– (*n* = 6)	WT (*n* = 8)	Shn3–/– (*n* = 8)	Inter action	geno‐ type	Age
OLS‐porosity (% )	1.4	**1.7** c	0.9 e	**1.2** c ^,^ e	0.7 d	0.9 d	ns	<.001	<.001
	(0.4)	**(0.4)**	(0.1)	**(0.1)**	(0.1)	(0.1)			
OLS‐density (nb./mm^2^)	603	629	467 d	519 e	474 d	509 e	ns	ns	<.001
	(112)	(79)	25	16	(45)	29			
OLS‐area (μm^2^)	23.8	**27.7** a	19.5 e	**23.9** b ^,^ e	15.6 d ^,^ g	**18.8** ^**b**,^ d ^,^ f	ns	<.001	<.001
	(2.3)	**(3.6)**	(0.9)	**(1.4)**	(2.1)	**(1.8)**			
OLS‐perimeter (μm)	21.3	22.1	19.5 e	**21.0** b	17.6 d ^,^ g	**19.0** b ^,^ d ^,^ g	ns	<.001	<.001
	(0.8)	(1.5)	(0.3)	**(0.8)**	(1.2)	**(1.0)**			

Data are mean (SD). *n* = number of studied mice. In bold: data that are significantly different to WT at similar age (based on post hoc comparison). Significance by Tukey post hoc comparison following two‐way ANOVA is indicated by superscript letters.

a *p* = .001.

b *p* < .01.

c *p* < .05 versus WT.

d *p* ≤ .001.

e *p* < .01.

f *p* < .001.

g *p* < .01 18 months versus 4 months (all based on post hoc comparison).

### Collagen cross‐links ratio (pyr/divalent)

FTIRI analyses were performed on metaphyseal cancellous bone of mice at 6 weeks and 4 months of age. Two‐way ANOVA revealed no significant dependency of the collagen pyr/divalent cross‐link ratio on either age or genotype (Table 5).

**Table 5:** Collagen Cross‐Link Ratios of Cancellous Bone

	6 Weeks		4 Months		Two‐way ANOVA (*p* values)		
	WT (*n* = 7)	Shn3–/– (*n* = 7)	WT (*n* = 4)	Shn3–/– (*n* = 6)	Inter action	geno type	Age
Pyr/divalent	0.34	0.32	0.34	0.41	.493	.362	.119
	(0.09)	(0.09)	(0.23)	(0.09)			

Data are mean (SD). *n* = number of studied samples.

Pyr/divalent = collagen cross‐link ratio index (trivalent pyridinoline cross‐links divided by divalent ones).

## Discussion

In this study, the mechanical competence of whole bone and intrinsic bone material properties of Shn3–/– mice were assessed and compared with those of WT mice. We found the ultimate force under bending and compression significantly increased for femora and vertebrae, respectively. The increase in ultimate force under compression of vertebra and bending of femora is consistent with the increased vertebral cross‐sectional bone areas, as well as bone volume per tissue volume and the increased cortical area and thickness, respectively, observed for the Shn3–/– mice. However, the remarkable increase in mechanical performance also suggests that the excessively formed bone material in these mice is not of compromised quality. This is in clear contrast to previous studies on sodium fluoride treatment in osteoporotic patients where the strong anabolic effect led in fact to a strong increase in vertebral bone mass, but not in a reduction of vertebral fractures.^25^, ^26^ Bone material formed during sodium fluoride treatment revealed a disturbed and highly heterogeneous mineralization distribution of the bone matrix with partly increased and partly deficient mineralization.^27^ In the present study, the improved mechanical performance remained after normalization for the change in estimated geometry (evidenced by the higher apparent strength).

The change in the volume of mineralized bone in the distal femoral metaphyseal region with age was highly dependent on the genotype. It is interesting that in the Shn3–/– mice the bone volume is increasing, especially from 6 weeks to 4 months, whereas the C57BL/6 WT mice lose bone mass with age, in agreement with the results of Glatt and colleagues.^28^ Previous histomorphometric outcomes revealed a fivefold increase in bone formation rate based on both increased mineralizing surface and mineral apposition rate in Shn3–/– mice, concurrently with unaltered osteoblast surface. The number of osteoclasts was comparable in Shn3–/– and WT skeletal tissue by histomorphometric analysis,^2^ although another publication reported that the serum markers of bone resorption were significantly reduced in young (6‐week‐old) Shn3–/– animals compared with WT littermates,^4^ further explaining the apparent positive net balance between bone formation and resorption. The ongoing overproduction by the osteoblasts and decreased resorption by the osteoclasts led to an osteosclerotic/osteopetrotic phenotype in the older Shn3–/– mice. The 18‐month‐old Shn3–/– animals exhibited a nearly filled‐up marrow space and a considerably increased cortical width compared with WT.

BMDD outcomes showed a normal degree of mineralization in the youngest study group, but a higher degree of bone matrix mineralization in the full‐grown Shn3‐deficient mice compared with WT animals. Based on the established computer modeling of the BMDD,^23^ we would expect a lower degree of mineralization in Shn3/‐. As previously shown by experimental data and computer modeling,^8^, ^23^ two processes are essential in the determination of BMDD: (1) the mineralization kinetics, ie, the time course of mineral accumulation in newly formed osteoid deposited by the osteoblast, which starts with an initial rapid phase of primary mineralization up to about 70% of final mineral content within days, followed by the slower phase of secondary mineralization achieving full mineralization within months^9^, ^29^, ^30^, ^31^; and (2) the bone turnover rate that determines the fraction of bone volume, which is apposed and/or replaced by new bone in a certain time (eg, year) because of bone (re)modeling.^8^ Based on the latter, a higher modeling/remodeling rate will reduce both tissue age and CaMean and vice versa.^8^ This is valid for the case of balanced bone formation and resorption (ie, constant bone volume). However, in the present work, the Shn3–/– exhibited a large increase in bone volume, thus the computer model needed to be modified. Unlike previous applications, trabecular bone volume was not set constant, but was allowed to increase rapidly with time in the present work. This modified computer model showed that the average calcium concentration of bone (CaMean) first decreases and subsequently increases with increasing bone volume (ie, increasing animal age). Unfortunately, the drop in CaMean occurs at an age not included in the present study. At the available ages, the computer model predicts the similarity of CaMean at 6 weeks and an increased CaMean at 4 and 18 months in line with the experimentally measured BMDD. Our findings suggest that in a case with large increases in bone volume (in Shn3–/–, trabecular bone volume increase is about twofold at 6 weeks and about eightfold in full‐grown animals), the average degree of mineralization can be similar or even higher compared with WT bone, solely caused by the increase in bone volume. This can be explained by the fact that the increase of bone volume causes an overall higher average tissue age, enabling a longer period of secondary mineralization. Interestingly, high bone volume accompanied by increased bone matrix mineralization was also observed previously in mice overexpressing the transcription factor Fra‐1,^19^ as well as in sclerostin‐antibody‐treated WT mice.^32^

Using qBEI images, we also measured the characteristics of the 2D sections of osteocyte lacunae in femoral midshaft bone from the study groups and observed significant differences between Shn3–/– and WT at all studied ages. The OLS porosity was higher, mainly because of a larger mean OLS area in the Shn3–/– mice. Apart from these differences in genotype, we also observed changes in OLS characteristics as a result of age. Cortical bone from younger animals showed larger OLS porosity and OLS area than that from older animals. This may be explained by the presence of remnants of primary bone in the cortex of younger mice. These remnants of primary bone have different OLS morphology (Fig. 1, inset with detail); however, they diminish during the modeling process of the femur in the WT. In contrast, in a Shn3‐deficiency, we found such remnants of primary bone in the full‐grown animals as well. This is likely caused by the altered modeling, and is in line with the previous report of decreased osteoclast activity in Shn3‐deficiency.^4^ However, the differences in OLS characteristics are not exclusively caused by the presence or absence of such primary bone remnants. We have compared OLS characteristics separately in cortical lamellar bone between the genotypes (data not shown) and found similarly increased OLS porosity, OLS area, and OLS perimeter in Shn3‐deficient bone in line with the results obtained from the entire cortex. The reason for differences in the morphology of the osteocyte lacunae remains unknown as no information on osteocyte activity or viability in Shn3‐deficiency is available.

Apart from the mineral, the second essential component of the bone material is its organic constituent, mainly type I collagen. Its most distinct feature in mineralized tissues is its cross‐linking chemistry, providing the fibrillar matrices with various mechanical properties.^17^ Cross‐link type and relative amounts depend on tissue age, bone turnover, and environmental factors.^33^ Studies suggest that altered collagen cross‐links contribute to changes of bone mechanical performance independent of proper bone mineralization.^24^, ^34^, ^35^, ^36^ Furthermore, in patients with idiopathic osteoporosis, increased bone fragility was found associated with a higher collagen cross‐links ratio.^37^ In this context, the similarity of collagen cross‐linking in the Shn3‐null and WT mice is an essential finding for the mechanical properties of the bone material.

We conclude that the bone volume under Shn3–/– deficiency is highly increased and both femora and vertebrae have higher ultimate force in mechanical testing. Taking the geometry of the femora and vertebrae into account, the estimated apparent strength of Shn3–/– bone remains higher than that of WT mice, suggesting no deterioration of the bone material properties in Shn3‐deficiency. No indications for changes in the mineralization kinetics are observed. The increased degree of bone matrix mineralization is likely caused by a larger bone volume/area in the Shn3‐deficient mice, a hypothesis that is confirmed by computer modeling. If one succeeds in regulating the excessive increase in bone volume, the inhibition of Shn3 expression or activity might be a promising target for the development of antiosteoporotic drugs.

## Disclosures

All authors state that they have nothing to disclose.
